# Sex-Related Differences in Short-Term Outcomes after Mobile VA-ECMO Implantation: Five-Year Experience of an ECMO Retrieval Program

**DOI:** 10.3390/life12111746

**Published:** 2022-10-31

**Authors:** Ihor Krasivskyi, Borko Ivanov, Johannes Vehrenberg, Kaveh Eghbalzadeh, Stephen Gerfer, Christopher Gaisendrees, Elmar Kuhn, Anton Sabashnikov, Navid Mader, Ilija Djordjevic, Thorsten Wahlers

**Affiliations:** 1 Department of Cardiothoracic Surgery, University Hospital Cologne, 50937 Cologne, Germany; 2 Department of Cardiothoracic Surgery, Helios Hospital Siegburg, 53721 Siegburg, Germany

**Keywords:** sex, cardiogenic shock, VA-ECMO

## Abstract

Veno-arterial extracorporeal membrane oxygenation (VA-ECMO) represents an increasingly used method for circulatory support. Despite the ongoing research, survival following VA-ECMO therapy remains low. Sex-related differences might impact the outcome of therapeutic measures. We aimed to compare all-cause mortality among female and male patients who underwent VA-ECMO as a bridge to recovery investigating sex-related differences. From January 2015 until August 2020, 87 patients were supported by VA-ECMO as a part of our out-of-center mobile ECMO program. In order to analyze sex-associated differences in early clinical outcomes, patients were divided into two sex categories: men (*n* = 62) and women (*n* = 25). All relevant data (in-hospital mortality, ICU and hospital stay, renal failure requiring dialysis, lung failure, bleeding, stroke and septic shock) were analyzed retrospectively after the extraction from our institutional database. Mean age of the study population was 53 ± 14 years. Mean EuroSCORE II predicted mortality was 6.5 ± 3.7. In-hospital mortality rate was not significantly lower in the female group (58.3%) vs. the male group (71.2%), *p* = 0.190. The mean length of ICU and hospital stay was 9 ± 11 in the male group vs. 10 ± 13 in the female group, *p* = 0.901, and 10 ± 12 (male group) vs. 11 ± 13 (female group), *p* = 0.909, respectively. Renal failure requiring hemodialysis (36.2% (males) vs. 28.6% (females), *p* = 0.187) was comparable between both groups. Respiratory failure was diagnosed in 31 (56.4%) male vs. 8 (34.8%) female patients, *p* = 0.068, while 16 (28.6%) male vs. 3 (13.0%) female patients (*p* = 0.118) suffered from septic shock. Based on our data, there were no sex-specific outcome discrepancies in patients treated with mobile VA-ECMO implantation.

## 1. Introduction

Veno-arterial extracorporeal membrane oxygenation (VA-ECMO) represents an increasingly used method for circulatory support. VA-ECMO in cardiogenic environments facilitates improvement of hemodynamic status and significant increase in tissue perfusion [1]. Despite the ongoing research, survival following VA-ECMO therapy remains low [2]. The impact of gender-related specifics on patients after VA-ECMO support is still controversially discussed [3]. However, gender-related differences might impact the outcome of therapeutic measures [2,3].

Several published studies demonstrated a significantly higher incidence of cardio–pulmonary resuscitation and subsequent extracorporeal cardiopulmonary resuscitation in male patients [4,5]. In general, sex-based differences are known to influence the development of cardiac diseases [6]. 

Most studies on VA-ECMO have focused on outcomes [7,8,9], and several of them reported that approximately 50% of patients supported by VA-ECMO were male [10,11]. Furthermore, male patients undergo VA-ECMO implantation more frequently during catecholamine-refractory cardiogenic shock or low-cardiac-output-syndrome [12]. On the other side, authors have reported higher long-term survival rates in reproductive-aged women than in same-aged men [13].

Therefore, we aimed to compare all-cause mortality among female and male patients who underwent VA-ECMO as a bridge to recovery as part of our mobile ECMO program.

## 2. Materials and Methods

The study was designed as a retrospective single center nonrandomized analysis of mobile VA-ECMO cohort. Over a 5-year-and-8-month period, from January 2015 until August 2020, a total of 87 patients underwent mobile VA-ECMO therapy (Cardiohelp, Maquet, Rastatt, Germany). In order to analyze sex-associated differences in early clinical outcomes, patients were divided into two categories divided by sex. This study included 62 men and 25 women.

### 2.1. ECMO-Center Protocol

Our mobile ECMO program is organized as previously described [14,15]. ECMO therapy was initialized corresponding to Extracorporeal Life Support Organization (ELSO) guidelines for VA-ECMO implantation [16]. General clinical examination and transthoracic echocardiography (TTE) were used for an on-site ECMO evaluation. ECMO therapy was implemented at peripheral hospitals and by patients transported to our center. 

Our anticoagulant protocol aimed for an activated clotting time (ACT) of 160–180 s and activated partial thromboplastin time (aPTT) of 60–80 s after intravenous infusion of unfractionated heparin to avoid potential thromboembolic events. In order to evaluate hemodynamic stability and possible weaning ability echocardiography, laboratory parameters and chest X-ray were performed. Moreover, heart function was evaluated daily using transesophageal echocardiography (TEE). 

ECMO weaning was initialized after haemodynamic stabilization. ECMO flow rate was decreased by 100–200 mL/h. Moreover, lactate and urine output was assessed hourly. ECMO removal was feasible when TEE showed partial or full recovery under 2.0 L/min ECMO support without increasing lactate concentration in the blood and decreasing urine output. All patients assumed suitable for weaning underwent surgical explantation of ECMO cannulas. 

### 2.2. Data Collection 

All relevant data were analyzed retrospectively after extraction from our institutional database and were collected on standardized forms and entered into a computerized database. The variables evaluated included such parameters as: patient demographic (age, sex, BMI, EuroSCORE II), patients’ status before ECMO support, laboratory parameter (creatinine, urea, aspartate aminotransferase (AST), alanine aminotransferase (ALT), platelet count) and early outcome data (in-hospital mortality, ICU and hospital stay, renal failure requiring dialysis, lung failure, bleeding, stroke and septic shock).

### 2.3. Outcome Analysis 

In this research we highlighted primary and secondary outcomes. The primary outcome in our study was in-hospital mortality after VA-ECMO therapy. Secondary outcome parameters were acute renal failure requiring dialysis, acute respiratory failure, bleeding, limb ischemia, septic shock, stroke, length of intensive care unit (ICU) and in-hospital stay.

### 2.4. Ethics

The study was conducted in accordance with the Declaration of Helsinki (as revised in 2013). The Ethics Committee of the Medical Faculty of the University of Cologne stated that we are exempted from applying for ethical approval, as under German law no separate ethics application or statement of ethical approval by the local ethics committee are required for performing purely retrospective clinical studies.

### 2.5. Statistical Methods

Statistics was performed using Student’s *t*-test or Mann–Whitney U test, each depending on whether continuous variables are normally distributed or not, and the chi-squared test was used for categorical variables (confidence internal for the difference of two means). Continuous variables are expressed as mean ± standard deviation (SD). Categorical variables are presented as percentage of the sample. Fisher exact test was performed when the minimum expected count of cells was <5. The optimal cut-off values were defined as the values that provided highest sensitivity and specificity. A *p*-value < 0.05 was considered to be significant. Statistical analysis was performed using Statistical Package for Social Sciences, version 28.0 (SPSS Inc., Chicago, IL, USA).

## 3. Results

**Figure 1:**
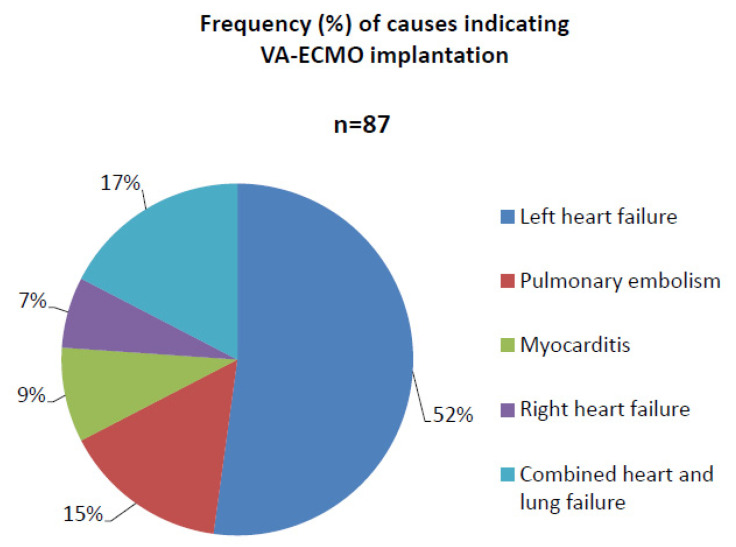
Frequency (%) of all causes due to cardiogenic shock before VA-ECMO implantation.

Generally, a total of 87 (*n* = 62 male group, *n* = 25 female group) patients underwent VA-ECMO therapy. Cardiogenic shock with left heart failure was the main cause (52%) indicating VA-ECMO implantation (Figure 1). Other causes indicating VA-ECMO implantation were: combined acute heart and lung failure (17%), pulmonary embolism (15%), myocarditis (9%) and acute right heart failure (7%).

### 3.1. Demographic, Clinical Characteristics and Postimplantation Data

Demographic, clinical characteristics and postimplantation data of female and male patients are shown in Table 1. Comparing both sex groups, peripheral VA-ECMO (93.3% (male group) vs. 100% (female group), *p* = 0.248) was implanted in the most cases. Initial VA-ECMO flow (4.0 ± 1.8 L/m (male) vs. 3.7 ± 2.3 L/m (female), *p* = 0.770) and VA-ECMO duration (90.4 ± 83.8 h (male) vs. 100.3 ± 82.6 h (female), *p* = 0.947) did not differ between both groups. Inotropic support (82.2% in the male group vs. 72.0% in the female group, *p* = 0.696) was similar in both groups. While two male patients (3.4%) were treated with additional left ventricular venting utilizing Impella CP^®^, no female patients received concomitant Impella CP^®^ support (*p* = 0.515). Weaning of VA-ECMO was feasible in 24 (42.1%) male patients and 14 (58.3%) female patients (*p* = 0.137). VA-ECMO duration support was not significantly higher among female patients (100.3 ± 82.6 h) compared to male patients (90.4 ± 83.8), *p* = 0.947. Further, the initial RBC transfusion rate (19.0 ± 20.5 units (male group) vs. 18.2 ± 16.9 units (female group), *p* = 0.646) showed no significant difference between both groups.

### 3.2. Laboratory Parameter 24 and 48 h after VA-ECMO Implantation

Laboratory parameters 24 and 48 h after VA-ECMO implantation are shown in Table 2 and Table 3. Creatinine levels were significantly higher in the male group (2.5 ± 1.9 mg/dL) compared to the female one ((1.5 ± 0.7 mg/dL), *p* < 0.043). Mixed venous oxygen saturation (76.5 ± 13.0% (male group) vs. 68.1 ± 25.2% (female group), *p* = 0.004) and carbonic dioxide partial pressure (41.2 ± 13.4 mm/Hg (male group) vs. 48.8 ± 32.5 mm/Hg (female group), *p* < 0.001) differed significantly depending on sex. In addition, hepatic cell damage parameter AST (974 ± 1492 U/L vs. 1744 ± 3079 U/L, *p* = 0.004) and ALT (610 ± 1063 U/L vs. 1110 ± 2075 U/L, *p* = 0.004) were significantly higher in the female group. In contrast, bilirubin rate (1.8 ± 3.7 mg/dL vs. 0.7 ± 0.5 mg/dL, *p* = 0.033) was significantly higher among male patients. Further, platelet count differed significantly between both groups (50.4 ± 87.7 × 10^9^/L (male group) vs. 103.8 ± 158.6 × 10^9^/L (female group), *p* < 0.001). Lactate (9.9 ± 6.9 mmol/L (male) vs. 9.6 ± 7.3 mmol/L (female), *p* = 0.491) and pH (7.2 ± 0.4 (male group) vs. 7.2 ± 0.1 (female group), *p* = 0.842) values showed no differences between male and female patients over the first 24 h. After 48 h, oxygen partial pressure (138.4 ± 69.8 mm/Hg (male group) vs. 117.1 ± 28.6 mm/Hg (female group), *p* = 0.027) was significantly higher in male patients. In addition, urea rate (76.2 ± 42.9 mg/dL (male) vs. 55.7 ± 27.1 mg/dL (female), *p* = 0.035) was significantly lower among female patients. Furthermore, bilirubin rate (3.4 ± 4.5 mg/dL (male group) vs. 1.8 ± 1.2 mg/dL (female group), *p* = 0.027) remained significantly higher in the male group. Additionally, platelet count (30.3 ± 57.3 × 10^9^/L (male group) vs. 67.0 ± 101.3 × 10^9^/L (female group), *p* < 0.001) differed significantly depending on sex after 48 h.

### 3.3. Primary and Secondary Outcome Parameters

**Figure 2:**
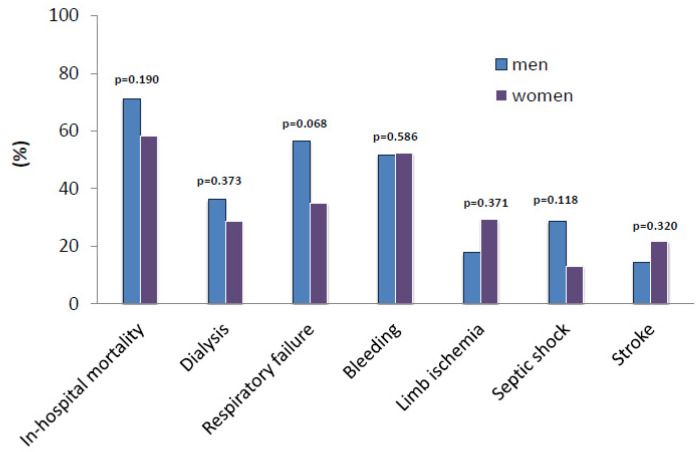
Primary and secondary outcome parameters of patients after VA-ECMO implantation due to cardiogenic shock.

Primary and secondary outcome parameters are summarized in Table 4. In-hospital mortality rate (Figure 2) was not significantly lower in the female group (58.3%) vs. the male group (71.2%), *p* = 0.190. The mean length of ICU and hospital stay was 9 ± 11 days (male group) vs. 10 ± 13 days (female group), *p* = 0.901 and 10 ± 12 days (male group) vs. 11 ± 13 days (female group), *p* = 0.909, respectively. Renal failure requiring hemodialysis (glomerular filtration rate (GFR) <15 mL/min, life-threatening hyperkalemia, refractory acidosis and hypervolemia causing end-organ complications) (36.2% (male group) vs. 28.6% (female group), *p* = 0.373) was comparable between both groups. Stroke (ischemic stroke or haemorrhagic stroke) and perioperative thromboembolic events (detected with computed tomography (CT) angiography) (8 (14.5%, male group) vs. 5 (21.7%, female group), *p* = 0.320 and 12 (21.8%, male patients) vs. 6 (26.1%, female patients) *p* = 0.446, respectively) did not differ significantly depending on sex. Overall, 17.9% (male group) vs. 29.2% (female group) of patients (*p* = 0.371) suffered limb ischemia (pain, pulseless, pallor, paralysis, paraesthesia and perishing with cold) after the procedure performed. Acute respiratory failure (PaO_2_/FiO_2_ ≤100 mmHg with PEEP ≥ 5 cm H_2_O) was diagnosed in 31 (56.4%, male group) vs. 8 (34.8%, female group) patients (*p* = 0.068), while 16 (28.6%, male group) vs. 3 (13.0%, female group) patients (*p* = 0.118) suffered from septic shock (persistent hypotension requiring vasopressors to maintain mean arterial pressure of 65 mm/Hg or higher and a serum lactate level greater than 2 mmol/L despite adequate volume resuscitation). In addition, hepatic failure (high levels of aspartate aminotransferase (AST), alanine aminotransferase (ALT), alkaline phosphatase (ALP), and total bilirubin with jaundice, dark urine color and abdominal swelling) (34.5% (male group) vs. 26.1% (female group) (*p* = 0.326) did not differ significantly depending on sex. Furthermore, bleeding rate (blood loss with a hemoglobin decrease of greater than 3 g/dL, any hemoglobin decrease of greater than 4 g/dL or transfusion of 2 units blood products or more) did not differ significantly (*p* = 0.586) between males (51.8%) and females (52.2%).

## 4. Discussion

In our study, we investigated sex-related differences regarding short-term outcomes after VA-ECMO therapy. According to our findings, sex has no impact on early outcomes after VA-ECMO therapy in patients after mobile ECMO implantation. 

There is a lack of studies investigating the effect of sex on short- and long-term outcomes of patients who underwent VA-ECMO implantation [2,3,9,11]. In general, several studies stated that female patients suffered from postoperative complications more often compared to men [17]. Likewise, women suffered more frequently from limb ischemia due to the anatomically smaller diameter of femoral vessels [18,19]. Smaller cannula sizes and an advanced cannulation technique could avoid such serious complications [3]. Moreover, female sex was an independent risk factor for haemorrhagic stroke among patients after VA-ECMO implantation due to cardiogenic shock [20]. Based on our data, no differences were obvious between male and female groups in contrast to the current literature. 

Several studies showed an increased rate of end-organ failure in male patients compared to female patients [21]. Acute renal failure is a feared complication and affected up to 80% of patients under VA-ECMO support [22]. Thrombosis, bleeding and coagulopathy were the most common risk factors for acute kidney injury requiring dialysis [22,23]. An increased creatinine level was shown to be an independent predictor of mortality in extracorporeal cardiopulmonary resuscitation (eCPR) patients [2,23]. Neugarten et al. [24] (2018) showed that female gender might be protective in prevention of renal failure in patients on ECMO. Furthermore, Gaisendrees et al. [2] (2021) found that female gender was associated with significantly lower risk for renal failure requiring dialysis after eCPR. In contrast, we found no differences between both groups. 

Our study showed no difference in stroke rates between female and male patients after VA-ECMO implantation. Likewise, various studies suggested that sex does not influence neurological outcomes in ECMO patients [25,26]. 

Moreover, we found no differences (*p* = 0.326) in acute hepatic failure between both groups. Similarly, Han et al. [25] stated no significant difference in extensive hepatic cell damage between male and female patients after eCPR. In contrast, further studies showed a significantly higher rate of liver damage in male patients compared to female ones [2,4,24,27]. On the contrary, we found a significantly higher rate of liver damage markers (AST (*p* = 0.004), ALT (*p* = 0.004)) in the male group. Moreover, we detected a significantly higher bilirubin level (*p* = 0.033) in the male group compared to the female group. Authors stated that hemolysis might be a responsible factor for elevated bilirubin level by patients on ECMO [28,29,30]. Moreover, Kaetner et al. [28] (2018) hypothesized that an elevated bilirubin level was a risk factor for a higher mortality rate after VA-ECMO implantation. Furthermore, authors highlighted that the increased bilirubin level (≥10mg/dL) and lactate (≥2.25 mmol/L) were associated with higher all-cause mortality [28]. Despite the significantly higher bilirubin rate (*p* = 0.033) in the male group, all-cause in-hospital mortality did not differ between both groups in our study. However, we could speculate that the similar mortality in male and female groups is related to the similar lactate level in both groups. Likewise, authors stated that all abovementioned risk factors (bilirubin and lactate) affect mortality, but only lactate showed a strong prognostic value [28,30].

Furthermore, female gender presented a significantly higher (*p* < 0.001) platelet count compared with male gender in our study. However, we found no significantly higher (*p* = 0.586) bleeding rate between both groups. Hermann et al. [31] (2019) stated that severe thrombocytopenia was associated with significantly higher (*p* < 0.001) risk for bleeding. Moreover, authors showed that bleeding events were more common than extracorporeal circuit clotting events [31]. Various studies showed that thrombocytopenia could subsequently increase the bleeding risk [32,33,34]. However, multiple further risk factors such as elderly age, central cannulation, delayed sternal closure and excessive anticoagulation were also associated with bleeding events [32,35]. Further studies showed that haemorrhagic complications due to thrombocytopenia and platelet dysfunction could increase the mortality rate to 17% [32,35,36]. 

Despite advances in perioperative management and use of modern technologies, all-cause mortality after VA-ECMO implantation remains high [32,35,37,38,39]. Based on our study, in-hospital all-cause mortality rate was 67.5% in patients who underwent mobile VA-ECMO implantation. Moreover, no significant differences (*p* = 0.190) in the mortality rate between both groups were found in our study. Likewise, an all-cause in-hospital mortality rate did not differ between male and female groups after VA-ECMO implantation in further studies [2,3,40]. 

Analyzing data from our study, differences in procedural techniques, surgeon experience, patient selection and perioperative care should be taken into account. Thus, further prospective randomized studies are needed in the future for more accurate sex-related analysis of end-organ damage and its correlation with short-, mid- and long-term results after VA-ECMO implantation.

## 5. Conclusions

Based on our data, sex does not affect short-term outcomes after VA-ECMO implantation. Mortality rates were almost the same in both groups (*p* = 0.190). Secondary outcome parameters (ICU (*p* = 0.901) and in-hospital stay (*p* = 0.909), renal failure requiring dialysis (*p* = 0.187), respiratory failure (*p* = 0.068), bleeding (*p* = 0.586), stroke (*p* = 0.320) and septic shock (*p* = 0.118)) did not significantly differ between male and female groups. However, prospective randomized trials are needed to investigate the impact of sex differences on short-, mid- and long-term outcomes after mobile VA-ECMO implantation.

## Figures and Tables

**Table 1:** Sex-related demographic, clinical characteristics and postimplantation data (*n* = 87).

	Male (*n* = 62)	Female (*n* = 25)	*p*-Value
Age (years), mean ± SD	54.3 ± 13.8	54.7 ± 13.7	0.976
BMI (kg/m^2^), mean ± SD	27.2 ± 6.2	28.1 ± 8.0	0.370
EuroSCORE II (%), mean ± SD	7 ± 3	5 ± 3	0.390
Distance to patient (km), mean ± SD	22.8 ± 24.6	30.4 ± 25.9	0.841
Central ECMO, *n* (%)	4 (6.7%)	0 (0.0%)	0.253
Peripheral ECMO, *n* (%)	58 (93.3%)	25 (100%)	0.248
Implantation technique, PP, *n* (%)	50 (89.3%)	25 (100%)	0.117
Arterial canula (Fr.), mean ± SD	17.7 ± 1.2	17.0 ± 1.1	0.020
Venous canula (Fr.), mean ± SD	22.1 ± 1.2	21.6 ± 1.1	0.151
DPC canula (Fr.), mean ± SD	7.2 ± 0.7	6.8 ± 0.8	0.797
eCPR, *n* (%)	18 (30.0%)	7 (30.4%)	0.584
Initial ECMO flow, L/m, mean ± SD	4.0 ± 1.8	3.7 ± 2.3	0.770
ECMO duration, h, mean ± SD	90.4 ± 83.8	100.3 ± 82.6	0.947
Inotropic support, *n* (%)	51 (82.2%)	18 (72.0%)	0.696
IABP, *n* (%)	6 (10.3%)	0 (0.0%)	0.125
Impella CP^®^, *n* (%)	2 (3.4%)	0 (0.0%)	0.515
ECMO weaning, *n* (%)	24 (42.1%)	14 (58.3%)	0.137
RBC, *n*, mean ± SD	19.0 ± 20.5	18.2 ± 16.9	0.646
FFP, *n*, mean ± SD	10.1 ± 13.9	7.3 ± 9.5	0.207
Platelets, *n*, mean ± SD	1.8 ± 2.6	1.5 ± 1.8	0.302

DPC, distal perfusion cannula; PP, per punktura; ECMO, extracorporeal membrane oxygenation; IABP, intra-aortic balloon pump; eCPR, extracorporeal cardiopulmonary resuscitation; Impella CP^®^, circulatory support device; RBC, red blood cell, FFP, fresh frozen plasma; Fr., French.

**Table 2:** Laboratory parameters after VA-ECMO implantation (24 h) (*n* = 87).

	Male (*n* = 62)	Female (*n* = 25)	*p*-Value
MAP (mmHg), mean ± SD	57.7 ± 21.7	56.6 ± 23.9	0.349
CVP (mmHg), mean ± SD	10.0 ± 6.0	13.2 ± 7.9	0.730
SvO_2_ (%), mean ± SD	76.5 ± 13.0	68.1 ± 25.2	0.004
pO_2_ (mmHg), mean ± SD	151 ± 110	200 ± 149	0.115
pCO_2_ (mmHg), mean ± SD	41.2 ± 13.4	48.8 ± 32.5	<0.001
pH, mean ± SD	7.2 ± 0.4	7.2 ± 0.1	0.842
FiO_2_ (%), mean ± SD	79.5 ± 25.5	75.0 ± 33.6	0.133
Urea (mg/dL), mean ± SD	80.6 ± 57.1	57.3 ± 35.5	0.068
Creatinine (mg/dL), mean ± SD	2.5 ± 1.9	1.5 ± 0.7	0.043
Lactate (mmol/L), mean ± SD	9.9 ± 6.9	9.6 ± 7.3	0.491
Bilirubin (mg/dL), mean ± SD	1.8 ± 3.7	0.7 ± 0.5	0.033
AST (U/L), mean ± SD	974 ± 1492	1744 ± 3079	0.004
ALT (U/L), mean ± SD	610 ± 1063	1110 ± 2075	0.004
Hb (g/dL), mean ± SD	11.1 ± 2.7	10.1 ± 3.3	0.193
Hct (%), mean ± SD	34.1 ± 7.6	30.4 ± 8.3	0.497
WBC (10^9^/L), mean ± SD	5.3 ± 8.8	8.0 ± 10.8	0.066
Platelets (10^9^/L), mean ± SD	50.4 ± 87.7	103.8 ± 158.6	<0.001
CRP (mg/L), mean ± SD	68.4 ± 91.2	76.7 ± 108.9	0.483
Na (mmol/L), mean ± SD	142.1 ± 6.7	140.6 ± 5.7	0.160
K (mmol/L), mean ± SD	4.6 ± 0.8	4.4 ± 0.7	0.673
aPTT (s), mean ± SD	76.0 ± 38.5	90.7 ± 38.1	0.294

MAP, mean arterial pressure; CVP, central venous pressure; SvO_2_, mixed venous oxygen saturation; pO_2_, oxygen partial pressure; pCO_2_, carbon dioxide partial pressure; pH, potential of hydrogen; FiO_2_, fraction of inspired oxygen; AST, aspartate transaminase; ALT, alanine transaminase; Hb, hemoglobin; Hct, hematocrit; WBC, white blood cell; CRP, C-reactive protein; aPTT, partial thromboplastin time.

**Table 3:** Laboratory parameters after VA-ECMO implantation (48 h) (*n* = 87).

	Male (*n* = 62)	Female (*n* = 25)	*p*-Value
MAP (mmHg), mean ± SD	66.9 ± 11.2	64.8 ± 10.4	0.505
CVP (mmHg), mean ± SD	13.3 ± 9.8	9.8 ± 3.4	0.357
SvO_2_ (%), mean ± SD	73.3 ± 6.8	75.7 ± 7.6	0.994
pO_2_ (mmHg), mean ± SD	138.4 ± 69.8	117.1 ± 28.6	0.027
pCO_2_ (mmHg), mean ± SD	39.6 ± 5.3	38.2 ± 5.4	0.964
pH, mean ± SD	7.4 ± 0.1	7.4 ± 0.08	0.874
FiO_2_ (%), mean ± SD	60.8 ± 48.0	44.1 ± 23.1	0.284
Urea (mg/dL), mean ± SD	76.2 ± 42.9	55.7 ± 27.1	0.035
Creatinine (mg/dL), mean ± SD	2.7 ± 3.3	2.2 ± 2.1	0.830
Lactate (mmol/L), mean ± SD	4.6 ± 4.8	3.2 ± 2.8	0.304
Bilirubin (mg/dL), mean ± SD	3.4 ± 4.5	1.8 ± 1.2	0.027
AST (U/L), mean ± SD	1978 ± 3292	1889 ± 3011	0.751
ALT (U/L), mean ± SD	877 ± 1440	720 ± 1080	0.381
Hb (g/dL), mean ± SD	10.2 ± 1.2	10.6 ± 1.6	0.874
Hct (%), mean ± SD	28.8 ± 3.6	28.4 ± 3.4	0.679
WBC (10^9^/L), mean ± SD	3.7 ± 6.9	6.2 ± 7.6	0.246
Platelet(10^9^/L), mean ± SD	30.3 ± 57.3	67.0 ± 101.3	<0.001
CRP (mg/L), mean ± SD	111.9 ± 83.6	128.5 ± 91.3	0.952
Na (mmol/L), mean ± SD	144.1 ± 4.3	142.6 ± 5.0	0.781
K (mmol/L), mean ± SD	5.8 ± 0.7	4.6 ± 0.4	0.243
aPTT (s), mean ± SD	61.7 ± 29.1	63.9 ± 22.2	0.280

MAP, mean arterial pressure; CVP, central venous pressure; SvO_2_, mixed venous oxygen saturation; pO_2_, oxygen partial pressure; pCO_2_, carbon dioxide partial pressure; pH, potential of hydrogen; FiO_2_, fraction of inspired oxygen; AST, aspartate transaminase; ALT, alanine transaminase; Hb, hemoglobin; Hct, hematocrit; WBC, white blood cell; CRP, C-reactive protein, aPTT, partial thromboplastin time.

**Table 4:** Sex-related complications after VA-ECMO implantation (*n* = 87).

	Male (*n* = 62)	Female (*n* = 25)	*p*-Value
Stroke, *n* (%)	8 (14.5%)	5 (21.7%)	0.320
Thromboembolic events, *n* (%)	12 (21.8%)	6 (26.1%)	0.446
Bleeding, *n* (%)	29 (51.8%)	12 (52.2%)	0.586
Limb ischemia, *n* (%)	10 (17.9%)	7 (29.2%)	0.371
Limb ischemia requiring intervention, *n* (%)	5 (8.9%)	4 (16.7%)	0.441
Respiratory failure, *n* (%)	31 (56.4%)	8 (34.8%)	0.068
Hepatic failure, *n* (%)	19 (34.5%)	6 (26.1%)	0.326
Renal failure, *n* (%)	34 (61.8%)	11 (47.8%)	0.187
Dialysis, *n* (%)	17 (36.2%)	6 (28.6%)	0.373
Oxygenator failure, *n* (%)	1 (1.9%)	(0.0%)	0.701
SIRS, *n* (%)	22 (40.0%)	6 (26.1%)	0.182
Septic shock, *n* (%)	16 (28.6%)	3 (13.0%)	0.118
ICU stay (days), mean ± SD	9 ± 11	10 ± 13	0.901
Hospital stay (days), mean ± SD	10 ± 12	11 ± 13	0.909
Mortality rate (in-hospital), *n* (%)	42 (71.2%)	14 (58.3%)	0.190

ICU, intensive care unit; SIRS, systemic inflammatory response syndrome.

## Data Availability

Data are available on a special request.
